# Anti-Inflammatory Effects of Sulfated Polysaccharide from *Sargassum swartzii* in Macrophages via Blocking TLR/NF-Κb Signal Transduction

**DOI:** 10.3390/md18120601

**Published:** 2020-11-28

**Authors:** Thilina U. Jayawardena, K. K. Asanka Sanjeewa, D. P. Nagahawatta, Hyo-Geun Lee, Yu-An Lu, A. P. J. P. Vaas, D. T. U. Abeytunga, C. M. Nanayakkara, Dae-Sung Lee, You-Jin Jeon

**Affiliations:** 1 Department of Marine Life Sciences, Jeju National University, Jeju 690-756, Korea; tujayawardena@jejunu.ac.kr (T.U.J.); asanka@jejunu.ac.kr (K.K.A.S.); pramuditha1992@jejunu.ac.kr (D.P.N.); hyogeunlee92@jejunu.ac.kr (H.-G.L.); luyuan@jejunu.ac.kr (Y.-A.L.); 2 Department of Chemistry, University of Colombo, Colombo 3, Sri Lanka; prasannarokx@gmail.com (A.P.J.P.V.); thusitha@chem.cmb.ac.lk (D.T.U.A.); 3 Department of Plant Sciences, University of Colombo, Colombo 3, Sri Lanka; chandi@pts.cmb.ac.lk; 4 Department of Applied Research, National Marine Biodiversity Institute of Korea, Seocheon 33362, Korea; 5 Marine Science Institute, Jeju National University, Jeju Self-Governing Province 63333, Korea

**Keywords:** *Sargassum swartzii*, TLR/MyD88, NF-κB, MAPK, fucoidan

## Abstract

This study involves enzymatic extraction of fucoidan from *Sargassum swartzii* and further purification via ion-exchange chromatography. The chemical and molecular characteristics of isolated fucoidan is evaluated concerning its anti-inflammatory potential in RAW 264.7 macrophages under LPS induced conditions. Structural properties of fucoidan were assessed via FTIR and NMR spectroscopy. NO production stimulated by LPS was significantly declined by fucoidan. This was witnessed to be achieved via fucoidan acting on mediators such as iNOS and COX-2 including pro-inflammatory cytokines (TNF-α, IL-6, and IL-1β), with dose dependent down-regulation. Further, the effect is exhibited by the suppression of TLR mediated MyD88, IKK complex, ultimately hindering NF-κB and MAPK activation, proposing its therapeutic applications in inflammation related disorders. The research findings provide an insight in relation to the sustainable utilization of fucoidan from marine brown algae *S. swartzii* as a potent anti-inflammatory agent in the nutritional, pharmaceutical, and cosmeceutical sectors.

## 1. Introduction

Brown algae is a promising source of secondary metabolites with potent bioactive properties. Asian countries are renowned for the traditional usage of marine algae as medicinal ingredients. The high availability, active properties, and the low processing cost has intensified the light of attention towards seaweeds in the functional food industry [1]. *Sargassum* belongs to the class phaeophyceae and is well exploited for its medicinal aspects [2,3,4], further elaborating studies were continued to the biosynthesis of nanoparticles utilizing extracted polysaccharides [5]. Marine brown algae is an important source of sulfated polysaccharides. Considering its health benefits, marine polysaccharides are well known and candidates in the medicinal, nutraceutical and pharmaceutical sectors. Fucoidan is a sulfate containing polysaccharide and possesses complex formation with valuable biological properties and is particularly popular among Asian food culture. Though the structural properties of fucoidan vary form one specie to another, it is mainly composed of fucose and sulfate substituted to the backbone with galactose, mannose, xylose, and uronic acid insertions [6,7].

Bioactive components from macro algae are extracted using different techniques, such as organic extraction, water based enzymatic methods, and implementation of microorganisms in the digestion process. Among these, utilization of enzymatic hydrolysates of algal biomass is beneficial over other practices. The technique does not involve any toxic chemicals and converts water insoluble material into water soluble components. Comparably, enhanced yield and biological activities encourages the usage of the particular technique [8]. The present study extracted fucoidan from *Sargassum swartzii* using enzymatic methods and further purified via ion-exchange chromatography.

Inflammation is a defense mechanism which activate physiological and pathological immune system procedures. This helps to restore normal conditions in the pathogen invaded tissue structure and function. Though, excessive inflammation leads to chronic disorders such as rheumatoid arthritis, Alzheimer’s disease, and cardiovascular diseases [9]. During inflammation, abnormal cytokine production, reactive oxygen species (ROS) level increment, and inflammatory signal pathway mediator activation is observed. The production of inflammatory cytokines are mediated via the association of iNOS and COX-2. Nitric oxide (NO) is an important end product, reflecting inflammation which is regulated via iNOS. LPS is a Gram-negative bacterial endotoxin that could activate macrophages via signaling pathways such as MAPK comprising of p38, ERK1/2, and JNK. Further, MAPKs are reported to be involved in the activation of NF-κB transcription [10]. Thus, suppression of the above mediators including pathway proteins are important in the prevention of inflammatory responses. 

Sulfated polysaccharide from *Sargassum* species has received much attention for its anti-inflammatory potential. Sanjeewa et al. (2019) reported isolation of fucoidan from *Sargassum horneri*. It revealed, LPS stimulated inflammation inhibition via NF-κB and MAPK pathways in macrophages. The researchers have further suggested the utilization of the particular species in Jeju island for potential development of polysaccharide based functional food material [11]. High molecular weight sulfated polysaccharides rich in fucose from *Sargassum* sp. was published to maintain anti-inflammatory potential. This alleviated NO production as well as pro-inflammatory cytokine production in macrophages [12]. *Sargassum hemiphyllum* sulfated polysaccharide was investigated by Hwang et al. (2015), with regard to its arachidonic acid-induced animal models of inflammation. The polysaccharide reduced neutrophilic infiltration in inflamed ears according to the histological examinations. Both oral and topical applications were investigated in the study, hence suggested as a treatment for inflammatory diseases [13]. 

*S. swartzii* grows along the shallow waters of the southern coastal area (Hikkaduwa, 6.1395° N, 80.1063° E) of Sri Lanka. This seaweed was reported previously on its bioactive properties such as antibacterial, antioxidant [14], and anti-viral potentials [15]. Former publications indicated the usefulness of fucoidan as a dietary supplement in human disease prevention due to its non-toxic potential [16]. Despite number of publications on fucoidan purified from seaweed and its biological activities, molecular mechanisms of fucoidan isolated from *S. swartzii* from Sri Lanka has not been investigated in detail with particular focus on TLR/MyD88 mediated MAPK, NF-κB expressions derived anti-inflammation. Hence, the authors aimed to purify and characterize sulfated polysaccharides from *S. swartzii* and to study downstream signaling mechanism associated with LPS-activated macrophage inflammation. This would enhance the understanding of *S. swatzii* fucoidan, extending its utilization in the cosmeceutical and functional food sectors.

## 2. Results

### 2.1. Chemical Composition of Algae Material and Purified Components

Carbohydrate content of the *S. swartzii* sample was the highest among all components indicating 62.78 ± 1.21%. Ash and moisture contents were recorded as 17.74 ± 1.24%, 4.82 ± 0.84% respectively. Lipid content was traceable (0.81 ± 0.17%), where protein content was 8.69% (Table 1).

**Figure 1:**
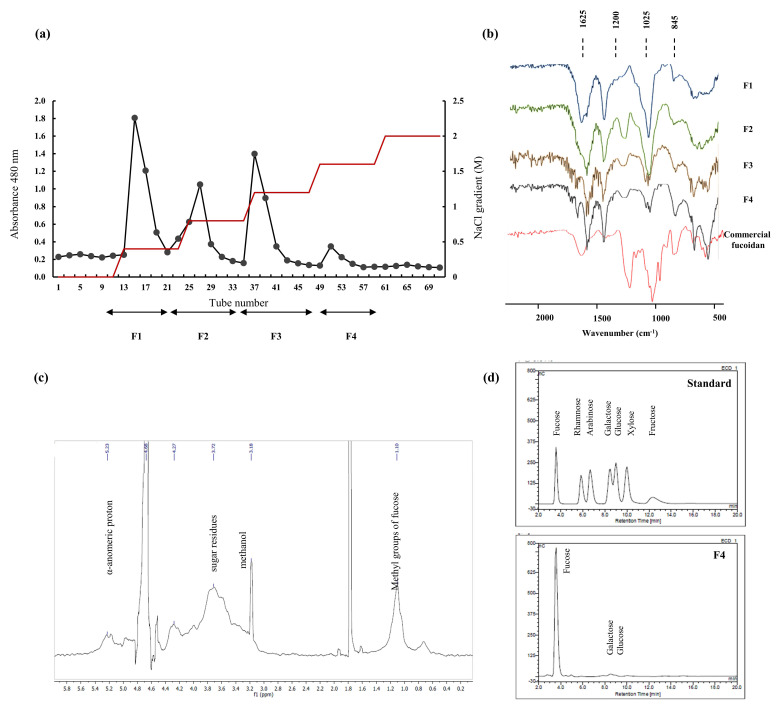
Purification and characterization of *S. swartzii*. (**a**) Crude polysaccharide purification through DEAE-cellulose anion exchange chromatography to obtain fractions of fucoidan, (**b**) FTIR analysis of each fraction against commercial fucoidan, (**c**) ^1^H NMR spectrum of F4, (**d**) monosaccharide composition analysis via HPAE-PAD spectrum (standard and F4).

Each polysaccharide fraction (Figure 1a) was analyzed for its chemical composition. Table 2, indicates the values of each in detail. Among all, polyphenol and protein contents were observed in lower amounts verifying the efficacy of the extraction procedure and DEAE column purification. The sulfate content exhibited an increasing trend while the polysaccharide yield was declining F1 through F4. Fraction F4 possessed 33.99 ± 0.17% sulfate, while F1 indicated 66.61 ± 4.32% polysaccharide.

Monosugar analysis results are tabulated in the Table 3. Fucose content was observed to be increasing with the anionic character of the polysaccharide. Other monosugars did not exhibit any particular trend. Minute amounts of galactose, glucose, and xylose were recorded.

### 2.2. Structural Characterization via FTIR and NMR Spectroscopy

FTIR spectrum of each fraction against commercial fucoidan was recorded and illustrated in the Figure 1b. The peaks of interest were observed in the range of 500 to 2000 cm^−1^. Polysaccharides are formed via glycosidic bonding among monosaccharide units. This is represented as a fingerprint region in the FTIR spectrum as 1035 cm^−1^ and is responsible for the C-O-C stretching vibrations. The significant sulfate amounts were reflected through the strong absorption band in the range of 1240–1255 cm^−1^ (S=O stretching). Sulfate group substitution at the C-4 position was expressed as a sharp peak at 840 cm^−1^ and a shoulder peak at 820 cm^−1^ (C-O-S). The bending vibrations of the H-O-H were observed in the 1600 cm^−1^ region which indicates traceable moisture content of the sample. C=O stretching vibrations of the carboxylic ester is designated as the 1700 cm^−1^ peak [17,18,19,20].

Differences in the relative expression of the above peaks were observed in the fractions. Fraction F4 was witnessed to be representing highest similarity with the commercial fucoidan FTIR. The results suggest sulfate substitution pattern from F1 through F4 and each fractions correlation with commercial fucoidan. 

Proton NMR spectrum of F4 (Figure 1c) was observed to possess characteristic peaks of fucoidan. Intense signals in the range of 5-5.5 ppm were an indication of the α-anomeric protons (H1 of α-L-fucopyranose). High field signal of 1.10 ppm was assigned as C6 methyl protons of L-fucopyranose. Peaks observed in the 1.6–2.0 ppm were due to methyl protons of the *O*-acetyl group [7,21,22]. Sugar residue protons are indicated as peaks in the range of 3.4–4.0 ppm [23]. The observed peaks verify chemical characteristic of fraction F4 as fucoidan. The ^13^C NMR was not satisfactory due to the polysaccharides structural heterogeneity and complexity.

### 2.3. Effect of Column Fractions on NO Production and Cell Viability in LPS-Induced Macrophages

**Figure 2:**
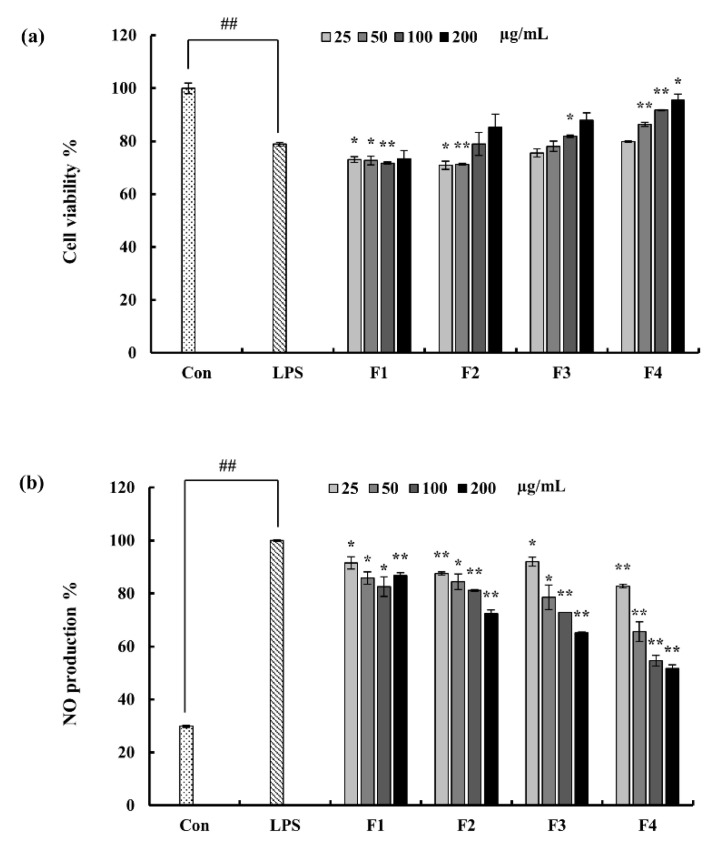
(**a**) Purified fractions from *S. swartzii* against LPS-induced cell viability, and (**b**) NO production in RAW 264.7 macrophages. Experiments were triplicated and results are indicated as means ± SD. Significantly different values from LPS treated group are represented as * *p* < 0.05 and ** *p* < 0.01 or ^##^ *p* < 0.01 against un-treated control.

The anti-inflammatory potential of the column fractions were evaluated in the LPS-stimulated macrophages. A key inflammatory mediator; NO production, is considered as a hint of inflammation. Accordingly with the results, LPS induced NO production was significantly inhibited by the fraction F4 in a dose-dependent manner. Even though fraction F3 has close potential to F4, considering the recovery of cell viability which was declined due to LPS, F4 was selected as the fraction with significant potential. Hence, F4 was subjected to further experiments (Figure 2).

### 2.4. Assessed Inflammatory Mediators

#### 2.4.1. Potential of F4 to Inhibit PGE_2_ and Pro-Inflammatory Cytokine Secretion

**Figure 3:**
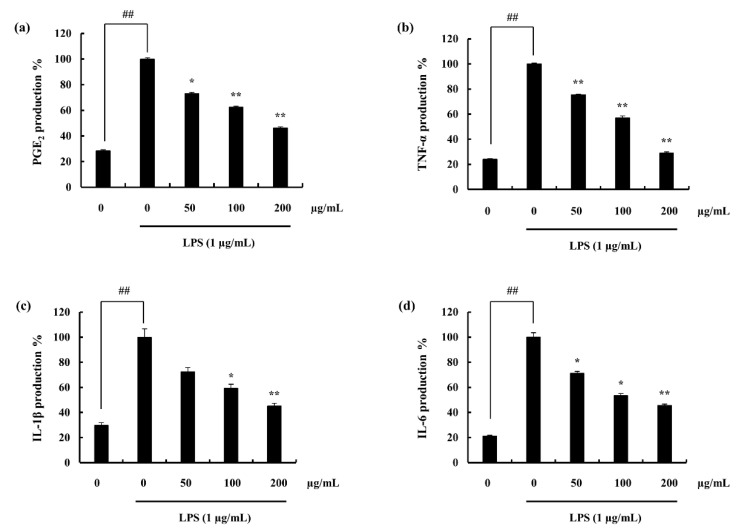
Effect of F4 on LPS induced (**a**) PGE_2_ and pro-inflammatory cytokine (**b**) TNF-α, (**c**) IL-1β, and (**d**) IL-6) expression of RAW 264.7 macrophages. Experiments were triplicated and results are indicated as means ± SD. Significantly different values from LPS treated group are represented as * *p* < 0.05 and ** *p* < 0.01 or ^##^ *p* < 0.01 against un-treated control.

**Figure 4:**
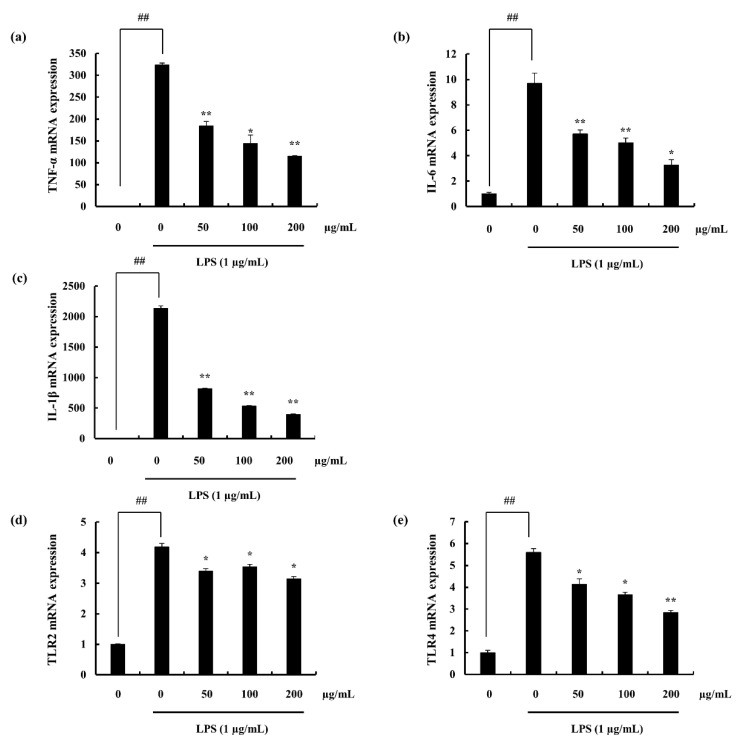
Gene expression levels analyzed as relative mRNA content in LPS-stimulated RAW 264.7 macrophages (**a**) TNF-α, (**b**) IL-6, (**c**) IL-1β, (**d**) TLR2, and (**e**) TLR4). The mRNA expression levels were measured via RT-qPCR techniques. Experiments were triplicated and results are indicated as means ± SD. Significantly different values from LPS treated group are represented as * *p* < 0.05 and ** *p* < 0.01 or ^##^ *p* < 0.01 against un-treated control.

ELISA results indicate the stimulation of the macrophages with LPS, increased the expression of each pro-inflammatory mediator. Pre-incubation with F4 successfully downregulated the PGE_2_ and pro-inflammatory cytokine levels. Furthermore, these inhibitions were dose-dependent, (Figure 3a–d). In addition, the results obtained via ELISA were solidified through the gene expression analysis, (Figure 4a–c).

#### 2.4.2. iNOS and COX-2 Expression Suppression and NF-κB Translocation Inhibition Ability of F4

**Figure 5:**
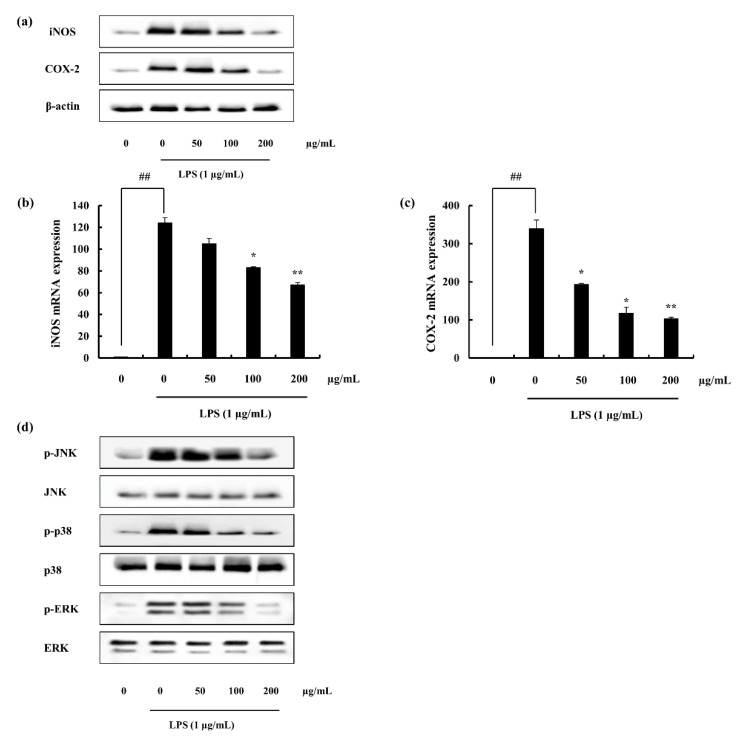
Effect of F4 against LPS-induced macrophages, (**a**) Western blot analysis of iNOS and COX-2, mRNA expression of (**b**) iNOS and (**c**) COX-2, (**d**) MAPK protein levels analyzed via Western blotting. Experiments were triplicated and results are indicated as means ± SD. Significantly different values from LPS treated group are represented as * *p* < 0.05 and ** *p* < 0.01 or ^##^ *p* < 0.01 against un-treated control.

iNOS; a major contributor in the inflammation via its impact on the NO production through acting as a catalyst was evident to be suppressed with the pre-treatment of F4. Moreover, COX-2 is important in the production of PGE_2_ via arachidonic acid pathway, was significantly declined by the action of F4. Both Western blotting analysis (Figure 5a) and gene expression assessment (Figure 5b–d) supports above facts.

**Figure 6:**
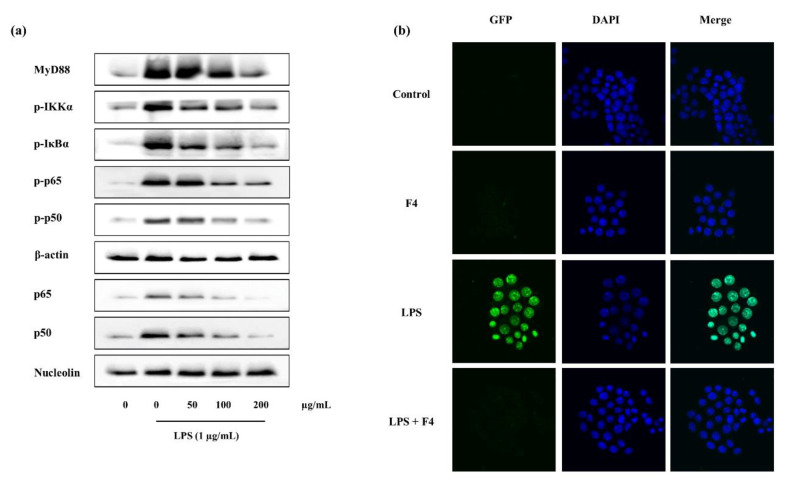
Influence of F4 analyzed over MyD88 mediated NF-κB pathway proteins, (**a**) Western blot results of pathway proteins, (**b**) nuclear translocation of NF-κB (p65) through immunofluorescence analysis, in RAW 264.7 macrophages.

NF-κB nuclear translocation was experimented via immunofluorescence methods. NF-κB which is composed of p50 and p65 is phosphorylated and translocated to the nucleus during inflammation to transcribe inflammatory related genes. Accordingly with the results, during LPS stimulation the translocation was significantly increased while pre-incubation with F4 declined it, (Figure 6b). These results were further confirmed with the Western blotting (Figure 6a). The phosphorylated levels of p50 and p65 were significantly declined with the F4 pre-treatment. Nucleus p50 and p65 levels which were increased due to the stimulation of LPS, were successfully down-regulated via F4 treatment, (Figure 6a).

#### 2.4.3. F4 Inhibits MAPK Phosphorylation Induced via LPS

The anti-inflammatory potential of F4 was further investigated with the analysis of MAPK pathway proteins (p38, JNK, ERK). In accordance with the results, LPS stimulation enhances the phosphorylation of MAPK proteins, where F4 treatment significantly declined it, (Figure 5d). Among assessed MAPKs, results imply the ability of F4 to act as an anti-inflammatory agent, (Figure 5d).

#### 2.4.4. Potential of F4 to Modulate the Activity of TLR-2/4 and MyD88 Dependent NF-κB Expression

TLR 2 and 4 mRNA levels were analyzed via RT-qPCR techniques. Macrophage TLR 2/4 levels were observed to be significantly inclined with the LPS stimulation. Pre-treatment with different concentrations of F4 down-regulated the expression of it. Further this was statistically proven to be significant. The mechanism of action, of blocking TLR was not studied during this research and requires further work to evaluate that, (Figure 4d,e).

The MyD88 dependent NF-κB proteins including, MyD88, IKK-α, IκB-α, p50, p65, and respective phosphorylated components were analyzed via Western blotting. Results indicate MyD88 levels to be increased with ascending F4 concentrations. Though the phosphorylated levels of IKK-α and IκB-α were increased with LPS stimulation, potential of F4 was sufficient to down-regulate them. Phosphorylation of p50 and p65 expressed similar trend with F4 pre-incubation, (Figure 6a).

## 3. Discussion

Exploration of marine organisms has become a topic of growing concern. Among them marine macro algae has become a source of sustainable bioactive secondary metabolites as well as a nutritional food component [24]. Studies have been numerously conducted on macro algal bioactive components and its bioactive potentials; polysaccharides [25], polyphenols [26], proteins, peptides [24], and sterols [27]. The brown algae of interest in this study was *Sargassum swartzii,* a member of the family *Sargassaceae*. This is abundant in the warm subtidal waters of Sri Lanka, along the coasts of Hikkaduwa. Earlier reports show investigation of sulfated polysaccharides of *S. swartzii* against antioxidant activity [14], and anti-viral activity [15]. Present study was conducted aiming purification of fucoidan from *S. swartzii* and its anti-inflammatory potential in LPS stimulated RAW 264.7 macrophages. 

Brown algal cell wall mainly contain three types of polysaccharides; cellulose, alginate, and fucoidans [28]. Fucoidan; a sulfated polysaccharide which belong to the fucans and is composed of a fucose backbone. This was first isolated and studied by Kylin in 1913 [29]. The structure of the fucans vary not only among different species but also with in the same. The complex, heterogeneous structure of these lead to isolation of distinct fucans depending on the purification method implemented [25]. Earlier reports point out extraction of fucoidan using water or aqueous solvent systems. Due to the above explained structural complexity and heterogeneity, isolation of potentially active polysaccharides were not tranquil. Interferences from other polysaccharides as well as vivid algal bioactive metabolites were necessary to be removed. The extraction method instigated in the study step wisely contributed to a better purification of the sulfated polysaccharides. Initially, the depigmentation process removes polyphenols as well as lipid components via dissolving them in 95% ethanol. Formaldehyde incorporation (10%) to 95% ethanol, assisted the polyphenol polymerization, hence, further elimination of phenolic substances from the final polysaccharide precipitate [30]. Even though fucoidans are soluble in acidic conditions, another major component of brown algae; alginic acids are insoluble. This property is well exploited in the study to eradicate the interference from alginic acid. The extraction solution is made acidic and addition of CaCl_2_ facilitates the precipitation of alginic acid in the form of its calcium salt, consequently removed via centrifugation [15,31]. The interference of proteins are removed via alcalase enzyme digestion [17]. Sulfated polysaccharides were recovered in the end step by means of ethanol precipitation which was expedited via the lowered di-electric constant, and sulfate groups actively participate in the process [32]. The extracted sulfated polysaccharide SWP was then subjected to anion exchange column purification (DEAE-cellulose) with the intention of fractionating it depending on the sulfate abundancy. It successfully yielded 4 fractions and among them F4 represented the highest sulfate content verifying the accuracy of purification. DEAE anion exchange column is consisted of quaternary ammonium functional groups. The NaCl gradient facilitates the exchange of ions depending on the strength of columbic interactions. Thus, highest sulfate content fraction elutes at the end. Chemical composition analysis of each fraction attest for the purification proficiency of fucoidan leaving traceable amounts of polyphenols as well as proteins. The polysaccharide content was declined while the sulfates were inclined among the fractions from F1 through F4. Similar reports have been published earlier by Fernando et al. (2017) and Sanjeewa et al. (2019) [18,33].

The structural characterization was conducted assisting FTIR and NMR spectroscopy. FTIR spectroscopic analysis provided an image of the functional group availability and active group F4s’ similarity towards the commercial fucoidan. The sulfate group (S=O) which is common to all fucoidans was observed with an intense peak at 1240 cm^−1^. Further C-O-S was seen at 820 cm^−1^ and 840 cm^−1^. These were well aligned with the commercial fucoidan and the intensities as well suggested its similarity. Earlier reports from Bilan et al. (2006) [34], and Lim et al. (2014) [20] supports the findings of this research. The proton NMR analysis of F4, provided key peaks relevant to polysaccharides as well as fucoidan structure. Among them α-anomeric protons and methyl group protons of the sugar residues were prominent. Due to fucoidans structural complexity and heterogeneity more details of the bonding formation could not be attained and requires further purification procedures such as deacetylation and de-sulfation. Usov et al. (1998) has made efforts in the elucidation of the structure of fucans form *Saccharina latissimi* via NMR analysis. This was further assisted with ^13^C NMR as well as 2D NMR techniques with additional purification steps [35].

Both initial bioactivity and chemical characterization results influenced the selection of F4 fraction to move forward with, to study detailed pathway mechanisms in the research. Stimulation of RAW 264.7 macrophages via LPS significantly decreased the cell viability and was successfully restored with F4 treatment expressing its cytoprotective ability [17,33]. This result was evaluated against the NO production inhibition analysis where F2, F3, and F4 expressed potent ability. Amongst, F4 was much effective. FTIR analysis against commercial fucoidan explained F4 fraction well aligned with commercial sample. Abundant fucose content via monosugar analysis as well conveyed its potential [36,37,38,39]. Thus, F4 was selected for subsequent experiments. 

Nitric oxide provides information on many biological processes and synthesized by a family of nitric oxide synthases having L-arginine as the substrate. Production of the aforementioned component in exceeded quantities are witnessed during immunological reactions. This as well is involved in pathogenesis conditions such that, septic shock, cirrhosis states and in inflammation [40]. Earlier reports have evidenced the role of nitric oxide as a part of both acute and chronic inflammation. Ialenti et al. (1992), published on the degree of inflammation to be reduced via the treatment of inhibitors of nitric oxide synthases where presence of L-arginine increased it [41]. Farrell et al. (1992), reported that patients with rheumatoid arthritis and osteoarthritis have increased levels of nitrite concentrations in plasma and synovial fluid [42]. Nitric oxide is cytostatic and cytotoxic, further it can combine with oxygen derived radicals to produce components with amplified toxicity [43]. Thus, increased production of nitric oxide could bring out detrimental effects on the neighboring cells. The results of this study exhibits F4 to reduce nitric oxide production (Griess assay) as well as the inducible nitric oxide synthase expression (Western blotting and mRNA analysis). The trend was observed to be dose-dependent. COX is a membrane bound, heme-containing glycoprotein. Two isoforms of the COX are found; COX-1 and COX-2, which are similar in structure and catalytic activity. Although it was evident that COX-2 was the primary enzyme which control PGE_2_ synthesis via arachidonic acid pathway in the inflammation process [44]. F4 fraction was evident to be an inhibitor of COX-2 as well as PGE_2_ expression. The production and the release of pro-inflammatory cytokines by macrophages were reported by Nathan (1987) [45]. These substances induce the migration of immune cells out of the circulation to the area of inflammation as well as attract macrophages and neutrophils to destruct the invading species [45,46]. Waiters (1994), states that cytokine release and illness response is observed due to the bacterial cell wall (LPS), killed bacteria, tissue damage, and nerve damage stimulation [47]. In the present study, LPS induced macrophages expressed higher amounts of pro-inflammatory cytokine release (IL-1β, IL-6, and TNF-α). F4 fraction had potential to down-regulate the mentioned levels dose-dependently, inhibiting the continuous transduction of inflammatory signals which would lead to unhealthy effects.

Algae derived fucoidans have come to the light of attention recently with respect to its potential therapeutic ability. Specially, algal biodiversity is immense besides used as food and folk medicine. Though, the structure of polysaccharide which is highly dependent on the source as well as the administration methodology would be vital in determining its potential. An elaborative review report was recently published by Lin et al. (2020), regarding the molecular targets relating to the biological activities of fucoidans [48]. Fucoidan is described to be effecting inflammatory process at different stages, both in anti-inflammatory and immunomodulation models. However, this discussion will only emphasis on the anti-inflammatory effects of fucoidan from *S. swartzii* comparatively to the previous findings. Among topics of attention of the possible mechanism to downregulate inflammation, fucoidans potential to alleviate MAPK and NF-κB signaling pathways thus reducing pro-inflammatory cytokines were widely conferred. Fucoidan from *Fucus vesiculosus* was modified (methacrylated fucoidan), subjected to LPS-stimulated RAW 264.7 macrophages to assess its anti-inflammatory potential. The study revealed potential of modified fucoidan to act upon elevated CD86 levels to be similar to that of IL-10. Further, the bioactive component exhibited protective role against LPS and IFN-γ stimulated growth inhibition [36]. High molecular weight fucoidan from *F. vesiculosus* was assessed against a spectrum of biological activities by Pozharitskaya et al. (2020), stated its significant ability to inhibit COX-2 enzyme (IC_50_ 4.3 µg mL^−1^) with grater selectivity index compared to synthetic anti-inflammatory drug, indomethacin [37]. Fucose rich polysaccharide from *Saccharina japonica* was published recently on its in-vitro and in-vivo anti-inflammatory effect. Accordingly, the researchers purified and characterized fucoidan from *S. japonica*, where it successfully alleviated LPS-induced inflammation through NF-κB and MAPK routs. The ROS levels which affect cell death were as well reported to be declined in the zebrafish analysis model [38]. Manikandan et al. (2020), publishes on fucoidan from *Turbinaria decurrens* and its ability to reduce formalin induced paw edema in mouse model and LPS induced cytotoxicity in IC-21 macrophages. The researchers attributed fucoidans potential to be arbitrated via modulating antioxidant enzyme levels and NF-κB signaling [39].

Hydroxyl radical (OH^●^), hydrogen peroxide (H_2_O_2_), and superoxide (O_2_^●−^) are chemically reactive oxygen species (ROS) resulted as natural byproducts of metabolic activities [49]. Pathophysiological processes involve excessive ROS production associated with metabolic changes. ROS production in innate immunity is commenced with the immune recognition via pattern recognition receptors (PRRs) or metabolic sensors (MS) [50]. Three isoforms of nitric oxide synthase (NOS) is available in mammalian cells: iNOS, eNOS, and nNOS. NOS uncoupling due to cofactor tetrahydrobiopterin (BH4) or substrate L-Arg deficiencies lead to decline in NO output. Increased O_2_^●−^ production via direct electron transfer to free O_2_ is a result of NOS uncoupling. NADPH oxidase complex (NOX) is another ROS generating enzyme which unlike others, directly catalyze O_2_^●−^ and H_2_O_2_ production [49]. Further, an elusive review published recently, describes the interplay between multiple ROS generators leading to further activation of each system, resulting a positive feedback mechanism [49]. Warnatsch et al. (2017), comments on the excessive production of ROS via NOX activation in neutrophils as a response to microbes; where is caused activation of NF-κB signaling resulting IL-1β expression levels and augmented neutrophil recruitment [51]. Thus, it is well evident the dual role of ROS, where it could play beneficial as well as bring detrimental effects. Regular ROS levels sustain proliferation, though extreme conditions both at low and high ends influence damage in cellular and organelle level.

The activated NF-κB/Rel transcription family plays an important role in the induction of pro-inflammatory gene transcription [52]. This transcription factor exist in the cytoplasm in its inactive form and is regulated by IκB inhibitors. Further IκB phosphorylation over NF-κB activation is regulated via IKK. Hence, activation of IKK phosphorylates IκB, initiating the releasement of p50/p65 subunits from the cytoplasmic NF-κB-IκB complex to be translocated to the nucleus [53,54]. NF-κB is reported to play an important role in the regulation of the pro-inflammatory gene-expression such as TNF-α, IL-6, and IL-1β [55]. It was well evident in the study that, F4 fraction down-regulated the phosphorylation of cytoplasmic p50/p65 units reducing its potential to be translocated to the nucleus thus inhibiting inflammatory signaling cascade. Moreover, the results were verified via the immunofluorescence assay of NF-κB nuclear localization. Similar results were previously published by Sanjeewa et al. (2019) utilizing ethanol extract of brown algae *Sargassum horneri* [4]. The involvement of MAPKs, in the process of inflammation has been reported by several researchers [56,57]. The MAPK pathway is an evolutionary conserved mechanism and is stimulated via hormones, cytokines, receptor mediators (PAMPs, DAMPs), and due to the stress of the environment. The cell physiology is affected by the activation of the particular pathway exhibiting gene transcription, cell control, differentiation of cells, and protein biosynthesis [58]. Thus, the effect of the MAPKs (p38, JNK, ERK) were evaluated against LPS stimulated macrophages pre-treated with F4. Active fraction (F4) exerted significant anti-inflammatory potential via down-regulating MAPKs phosphorylation. Ability of fucoidan from marine algae to down-regulate inflammatory responses were earlier reported by a number of researchers [57,59]. TLR and its subcomponents share a Toll-interleukin receptor domain (TIR). MyD88 is one of the distinct molecules interact with TLRs. These components recruit IL-1 receptor associated kinases (IRAKs) to cascade downstream signals which eventually initiate transcription factors such that; AP-1, IRF-5, and NF-κB [60]. Hence, this study evaluates MyD88 and TLR2/4 for each components’ role in the inflammation of LPS stimulated RAW 264.7 macrophages. Results exhibit successful down-regulation of the MyD88 expression through Western blotting as well as TLR2/4 decline via gene expression analysis with the F4 treatment. TLR2/4 signal activation involvement in the inflammatory pathway was recently assessed against CCl_4_ treated mice in which results of the particular study explained its down regulation via a flavonoid (quercetin), supported inactivation of further down-stream signals such as NF-κB, MAPK, and inflammatory cytokines [61].

Environmental factors including temperature, pH, light intensity, nutrient availability, and precipitation influence plant physiology and biochemistry [62]. The seasonal dynamics of the floral and faunal density and the tropic status of water was assessed by Ansari et al. (2015), expressing its research importance [63]. The algal biodiversity and the growth is altered due to climatic changes. Extreme low and high changes in climatic factors were reported to be compelling the above [64]. Marine brown algae *Sargassum horneri* with regard to chemical composition with seasonal variation was analyzed by Murakami et al. (2011), where they revealed the chemical composition to be correlated to growth and maturity [65]. Early spring was suggested to do harvest, where it would express maximal values of mineral content and dietary fibers in the particular sampling location, Fukuoka, Japan. Present study discusses on the evaluation of selected marine algae specie in a given time of sampling. This was a tropical seaweed specie and the collection point experiences similar weather annually. Though, due to water current, precipitation, and other factors it would experience minute changes. It would be beneficial the study could be extended to the level of analysis of certain changes in future research endeavors.

## 4. Materials and Methods 

### 4.1. Materials

Enzymes used in the experiment (Celluclast and Alcalase) were purchased from Novo Co. (Novozyme Nordisk, Bagsvaerd, Denmark). Cell line (RAW 264.7 macrophages) was purchased from the Korean Cell line bank (KCLB, Seoul, Korea). Growth media DMEM (including FBS and antibiotics; penicillin and streptomycin) were purchased from the Gibco Inc., (Grand Island, NY, USA). ELISA commercial kits (TNF-α, IL-6, IL-1β, and PGE_2_) were obtained from the eBioscience, Inc (San Diego, CA, USA), BD Biosciences (San Jose, CA, USA) and R&D systems, Inc (Minneapolis, Min, USA). Antibodies required for the Western blot analysis were obtained from Santa Cruz Biotechnology (Paso Robles, CA, USA). Fucoidan standard, potassium bromide (FTIR grade), and 3-(4,5-dimethylthiazol-2-yl)-2,5-diphenyltetrazolium bromide (MTT) were purchased from Sigma (St. Louis, MO, USA). Further, all the chemicals used in the experiments were of analytical grade and were purchased from Sigma, unless otherwise specified.

### 4.2. Extraction of Crude Fucoidan

The sample of interest *Sargassum swartzii* was collected from the coasts of Hikkaduwa, Sri Lanka (6.1395°N, 80.1063°E) during January 2019. Sample was immediately washed with running water to remove sand and epiphytes. This was lyophilized and ground into powder prior using for extraction procedures. Powder sample (100 g) was immersed in 95% ethanol for initial depigmentation and was continued for three repetitions. Further, a solution of 10% formaldehyde in 95% ethanol was used to remove attached polyphenols. Excess formaldehyde was washed away using ethanol. The ethanol was totally evaporated and the powder was suspended in distilled water adjusting the pH (4.5) with 1M HCl. Celluclast enzyme was introduced to the solution (0.5% of the substrate) and the extraction was carried out for 24 h under shaking kinetics. The temperature and the pH of the solution were maintained at 50 °C and 4.5 respectively, during the extraction period. With the completion of extraction, enzyme was heat inactivated (boiling water bath, 95 °C, 10 min). The supernatant was clarified through filtration and successive centrifugation. The supernatant pH was adjusted to 8.0 and alcalase was introduced. This was incubated at 50 °C for 24 h with continuous shaking. Enzyme was inactivated similarly as above. The pH was brought back to slightly acidic value and an aqueous solution of CaCl2 was added; precipitating alginates in the form of its calcium salt. Calcium alginates were removed via centrifugation and the solution pH was re-adjusted to its neutral value. This was concentrated to 1/3 of its original volume through lyophilization. Polysaccharides were precipitated by the addition of 95% ethanol, the mixture was stirred and left at 4 °C for 8 h. Suspended polysaccharides were recovered through centrifugation and was dissolved in distilled water. This was lyophilized to obtain the crude polysaccharide and referred as *S. swartzii* polysaccharide (SWP) [17,18].

### 4.3. Purification of SWP by Anion-Exchange Chromatography

The DEAE-cellulose (GE Health care, Uppsala, Sweden) packed column was pre-equilibrated with sodium acetate buffer (50 mM, pH 5.0). SWP sample portion was dissolved in the same buffer, filtered and introduced to the column. The elution was done with a NaCl solution in the same buffer starting from 0.2 M increasing upto to 2.0 M concentration, step wisely. The column eluates were collected to tubes in equal volumes and the polysaccharide content was analyzed via phenol-sulfuric method. Tube fractions were pooled and lyophilized. Following, dialysis membranes (Spectra/Por from Sigma, St. Louis, MO, USA, 3.5 kDa) were used to remove the ionic contaminants. Dialysis process was repeated with DW replacement until the conductivity reached the value of DW.

### 4.4. Chemical Analysis

Methods specified by AOAC were referred in the analysis of carbohydrate, moisture, ash, protein, and lipid contents [66]. SWP as well as the four fractions were analyzed for its polysaccharide, polyphenol, protein, and sulfate contents. Polyphenols were assessed via Folin-Ciocalteu method [67], while phenol-sulfuric method was followed in the analysis of polysaccharide content [68]. Protein content was measured by Pierce BCA protein assay kit. BaCl2 gelation method was used for the sulfate content analysis [69].

### 4.5. Structural Characterization

#### 4.5.1. FTIR Spectroscopic Characterization

A Bruker FTIR, AlphaⅡ (Bruker, Germany) instrument was used to obtain the attenuated total reflectance Fourier transform infra-red (ATR-FTIR) spectrum in the 400–4000 cm^−1^ wave number range. Each spectra were analyzed and compared against commercial fucoidan.

#### 4.5.2. NMR Analysis

The active fraction F4 was analyzed using NMR spectroscopy (JEOL JNM-ECX400, 400 MHz, Tokyo, Japan). Sample was prepared via continuous deuterium exchange, and addition of deuterated methanol as an internal standard. Proton NMR spectrum was obtained with 64 scans.

#### 4.5.3. Monosaccharide Analysis

The sample fractions were acid hydrolyzed with 4M trifluoroacetic acid (TFA). This was then subjected to separation via CarboPac PA1 column (4.5 mm × 50 mm) and was detected with an ED_50_ Dionex Electrochemical Detector [70]. A standard mixture was injected in the similar manner and relevant calculations were conducted accordingly. Monosaccharide amount calculations were done without considering the undetected constituents (unhydrolyzed components, contaminants, and sulfates).

### 4.6. Cell Culture

#### 4.6.1. Maintenance of Cell Line

Macrophage cell line (RAW 264.7) was cultured in DMEM medium supplemented with 10% FBS and 100 IU/mL antibiotics (penicillin and streptomycin). Subculture was conducted periodically maintaining 70–80% confluency. Cells were exposed to experiments in its exponential growth phase. Cells were conditioned in a Sanyo incubator (Sanyo MCO–18AIC; Sanyo, Moriguchi, Japan) maintaining temperature (37 °C), humidified atmosphere and 5% CO_2_.

#### 4.6.2. Cytotoxicity and Cell Viability Assessment

Cells were plated with a cell density of 1 × 10^5^ cells mL^−1^ in 24 well plates and incubated 24 h. Following, the cells were treated with sample fractions including different concentrations and incubated further 24 h. Cytotoxicity was assessed via MTT assay [71]. Non-toxic range was identified and used for the successive experiments. For cell viability assessment against LPS stimulation, cells were plated similarly as above and samples were treated. After 1 h incubation period, cells were stimulated with LPS (1 µg/mL) and continued incubation for another 23 h. The cell viability was evaluated by MTT assay.

#### 4.6.3. Nitric Oxide Production Inhibition Potential

The nitric oxide (NO) release was taken as a measurement of nitrite production. Cells were plated as explained above, treated samples and induced with LPS. After subsequent incubation period, Griess assay was performed with equal volumes of cell supernatant and Griess reagent [72].

### 4.7. Evaluation of Mediators in the Inflammatory Pathway

#### 4.7.1. Assessing Pro-Inflammatory Cytokines

The plated cells were exposed to different concentrations of F4 sample and induced with LPS. After a complete incubation of 48 h, cell supernatants were retrieved, then analyzed for PGE_2_, TNF-α, IL-6, and IL-1β using commercial immunoassay kits, following the manufactures instructions.

#### 4.7.2. Western Blot Analysis

For Western blot analysis, initially the cells were plated and (2 × 10^5^ cells mL^−1^) and treated samples as similar way explained in above experiments. Cells were harvested, lysed and the proteins were extracted. The protein content was measured and standardized using BCA protein assay kit (Bio-Rad, Irvine, CA, USA). Electrophoresis was conducted in 12% sulfate-polyacrylamide gels. This was transferred to nitrocellulose membranes, and blocked with 5% skim milk. Primary antibodies were introduced and incubated. This was followed by the addition of HRP-conjugated secondary antibodies. The bands were developed via a chemiluminescent substrate (Cyanagen Srl, Bologna, Italy) and photographed (FUSION SOLO Vilber Lourmat system, Paris, France). Quantification was done using the ImageJ program [4,73]. iNOS, COX-2, and NF-κB including MAPK pathway related proteins were analyzed. Target protein expressions were normalized by β-actin with regard to the cytosol and nucleolin for nucleus.

#### 4.7.3. Nuclear Localization of NF-κB via Immunofluorescence

Cells were seeded in 8-well slides (Nunc, Rochester, NY, USA). Samples were treated, stimulated with LPS and incubated. Cells were then fixed with 4% paraformaldehyde (10 min). Followed by a washing step with phosphate buffer saline (PBS), (3 × 5 min). Then, permeabilized with 0.1% Triton-X-100 and was continued with another washing procedure same as above. Donkey serum (10%) was used for the cell blocking. The slides were incubated overnight with NF-κB p65 in donkey serum (1:200) at 4 °C. Secondary antibody was added and incubated at room temperature (2 h) and followed by washing steps. A 10 min incubation was maintained after the addition of nuclear stain (DAPI). Excess staining was successfully removed via PBS washing. The chamber glass slides were covered with coverslips and mounting medium was added (Fluor shieldTM histology, Sigma, St. Louis, MO, USA). Images were captured by confocal microscope (Carl Ziess, Oberkochen, Germany) [4,74].

### 4.8. Analysis of Gene Expressions Related To Inflammatory Pathway

#### 4.8.1. RNA Extraction and cDNA Preparation

Trizol reagent (Life Technologies, Carlsbad, CA, USA) was used to extract total RNA from the RAW 264.7 macrophages. The extraction procedure followed manufactures instructions. RNA purity was measured (µDrop plate, Thermo Scientific, Waltham, MA, USA) and produced cDNA via reverse-transcription with a first-strand cDNA Synthesis Kit (TaKaRa, Shiga, Japan Japan) following given instructions. (Primers are indicated in Table 4)

#### 4.8.2. Real-Time Quantitative PCR

cDNA amplification was conducted using the given primers. PCR cycling conditions followed the method described by Sanjeewa et al. (2019) [4]. Quantification of relative gene expressions were done accordingly with the method explained by Livak and Schmittgen (2001) [75]. GAPDH was used as an internal control to normalize the relative gene expressions.

### 4.9. Statistical Analysis

Each experimental assay was triplicated to obtain the final data. Data are expressed as mean ± standard deviation. Statistical analysis was performed via IBM SPSS statistics for Windows, version 20.0 program assisted with one-way ANOVA. Significant level was considered as *p* values less than 0.05 (*p* < 0.05).

## 5. Conclusions

The study purifies fucose containing polysaccharide (fucoidan), form the marine brown algae *S. swartzii* which was sampled from the coastal area of Hikkaduwa, Sri Lanka. The purification procedure implemented in the study resulted in the isolation of fucoidan fraction (F4) which had well aligned characteristics with commercial fucoidan. The anti-inflammatory potential of F4 was observed to be acting through the inhibition of inflammatory mediators such as iNOS, COX-2, NO, and pro-inflammatory cytokines. Further, nuclear transcription factors (p50/ p65) as well as MAPK signaling with TLR2/4 involvement was evident in the mechanism of action of F4. The research findings provide an insight in relation to the sustainable utilization of fucoidan from marine brown algae in the mediation, prevention and intervention of inflammatory diseases. Thus, fucoidan from *S. swartzii* could be successfully utilized as a potent anti-inflammatory agent in the nutritional, pharmaceutical and cosmeceutical sectors combined with further in vivo studies.

## Figures and Tables

**Table 1:** Proximate composition of general components in *Sargassum swartzii*.

Component	Composition (%)
Ash	17.74 ± 1.24
Moisture	4.82 ± 0.84
Protein	8.69 ± 0.46
Lipid	0.81 ± 0.17
Carbohydrate	62.78 ± 1.21

All results expressed as means ± SE, based on triplicated trials.

**Table 2:** Proximate composition of general components in *Sargassum swartzii*.

	Polysaccharide Content (%)	Sulfate Content (%)	Protein Content (%)	Polyphenol Content (%)
Crude fucoidan	66.61 ± 4.32	20.83 ± 0.47	2.57 ± 1.44	1.66 ± 0.71
F1	81.05 ± 4.48	13.48 ± 0.72	0.79 ± 0.36	0.66 ± 0.21
F2	70.95 ± 1.83	21.86 ± 0.26	0.53 ± 0.36	0.41 ± 0.16
F3	65.91 ± 1.99	25.89 ± 0.42	0.66 ± 0.18	0.35 ± 0.12
F4	60.98 ± 0.66	33.99 ± 0.17	0.41 ± 0.18	0.32 ± 0.15

All results expressed as means ± SE, based on triplicated trials.

**Table 3:** Proximate composition of general components in *Sargassum swartzii*.

	F1	F2	F3	F4
Fucose	23.86	69.37	74.05	82.46
Rhamnose	2.72	ND	0.74	ND
Galactose	20.59	4.14	2.65	3.19
Glucose	16.37	2.67	1.51	1.35
Xylose	18.06	3.82	1.79	ND
Others	18.40	20.00	19.26	13.00

ND-not detected.

**Table 4:** Sequence of the primers used in the study.

Gene	Primer Sequence
GAPDH	antisense; 5′-AAGGGTCATCATCTCTGCCC-3′ and sense, 5′-GTGATGGCATGGACTGTGGT-3′
TLR2	antisense; 5′-CAGCTGGAGAACTCTGACCC-3′ and sense, 5′-CAAAGAGCCTGAAGTGGGAG-3′
TLR4	antisense; 5′-CAACATCATCCAGGAAGGC-3′ and sense, 5′-GAAGGCGATACAATTCCACC-3′
IL-1β	antisense; 5′-CAGGATGAGGACATGAGCACC-3′ and sense, 5′-CTCTGCAGACTCAAACTCCAC-3′
IL-6	antisense; 5′-GTACTCCAGAAGACCAGAGG-3′ and sense, 5′-TGCTGGTGACAACCACGGCC-3′
TNF-α	antisense; 5′-TTGACCTCAGCGCTGAGTTG-3′ and sense, 5′-CCTGTAGCCCACGTCGTAGC-3′
iNOS	antisense; 5′-ATGTCCGAAGCAAACATCAC-3′ and sense, 5′-TAATGTCCAGGAAGTAGGTG-3′
COX2	antisense; 5′-CAGCAAATCCTTGCTGTTCC-3′ and sense, 5′-TGGGCAAAGAATGCAAACATC-3′
